# Bilateral Internal Carotid Arteries Occlusion: A Case Report

**DOI:** 10.31729/jnma.6877

**Published:** 2022-02-28

**Authors:** Prabhaw Upadhyaya, Prasanna Karki, Baburam Pokharel, Gopal Raman Sharma

**Affiliations:** 1 Department of Neurosciences, Division of Neurology, Nepal Mediciti, Sainbu, Bhaisepati, Laiitpur, Nepal; 2 Department of Neurosciences, Division of Neurosurgery, Nepal Mediciti, Sainbu, Bhaisepati, Lalitpur, Nepal

**Keywords:** *case report, internal carotid arteries*, *ischemic stroke*, *thrombolysis*

## Abstract

Bilateral internal carotid artery occlusion is a disease that is encountered rarely. We report a case of a 54 years old female smoker, who presented with acute onset right-sided limb weakness, facial deviation and slurring of speech. On computed tomography angiography occlusion of the bilateral internal carotid artery was seen and a high-flow collateral circulation was formed through the vertebrobasilar system. Computed tomography brain perfusion showed marked cerebral hypoperfusion on the left side. The patient was thrombolysed and kept on dual antiplatelet therapy. Post-medical treatment, motor power and speech significantly improved.

## INTRODUCTION

Incidence of unilateral internal carotid artery occlusion is a well-recognized entity, however, bilateral internal carotid artery occlusion is a rare phenomenon.^1-5^ Internal carotid artery occlusion usually represents the end-stage of progressive carotid arterial disease. The natural history of patients with internal carotid artery occlusion secondary to atherosclerosis is usually influenced by the occurrence of a stroke around the time of occlusion. Symptomatic patients with internal carotid artery occlusion remain at a significant risk for future strokes. Studies report states that patients with cerebral infarcts secondary to carotid thrombosis have a subsequent stroke risk of 8-12% per year.^2-4^

## CASE REPORT

**Figure 1:**
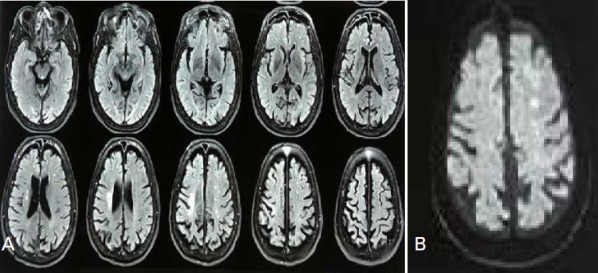
A) AXIAL Magneti c Resonance Imaging-Fluid-attenuated Inversion Recovery (MRI-FLAIR) showing lacunar infarction left MCA-ACA territory, B) AXIAL Diffusion-weighted Imaging (DWI) showed multiple punctate high signal intensity areas in the left frontal and parietal lobe at the ACA-MCA watershed zone (arrow).

**Figure 2:**
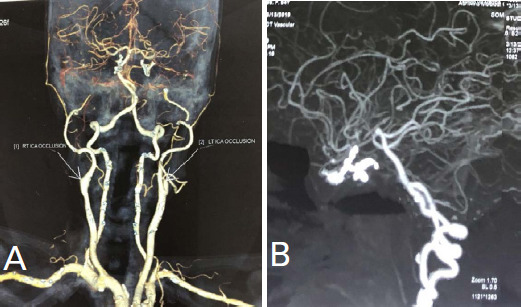
A) Computed tomography angiogram (CTA) demonstrating complete occlusion of bilateral internal carotid arteries just distal to common carotid arteries, B) Computed tomography angiogram (CTA) showing predominant cerebral vascular supply from posterior circulation.

**Figure 3:**
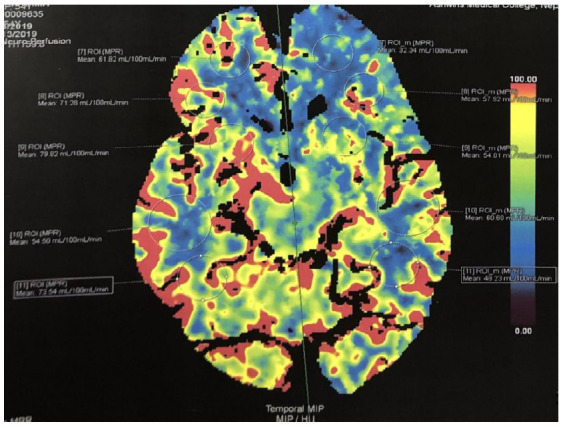
Computed tomography Perfusion showing hypoperfusion on the left ACA-MCA area.

A 54 years old lady presented with complaints of acute onset weakness of right upper and lower limbs, slurring of speech and right facial palsy, House-Brackmann classification: moderate dysfunction. She was normotensive and non-diabetic. She had an uneventful past medical history and used to smoke 10 cigarettes per day (pack year 17.5). On the physical examination, she was oriented but was slightly confused. Her right upper and lower limbs power were three according to the Medical research council scale (MRC scale). There was evidence of an ill-marginated heterogeneous fluid-attenuated inversion recovery (FLAIR) high signal intensity area in the left corona radiate (Figure 1A). Axial Diffusion-weighted Imaging (DWI) (Figure 1B) showed multiple punctate high signal intensity areas in the left frontal and parietal lobe at the anterior cerebral artery-middle cerebral artery (ACA-MCA) watershed zone. Computed tomography angiography (CTA) demonstrated that the bilateral internal carotid arteries (ICAs) were occluded and that the entire Willis circle was completely enhanced, with the vertebral-basal arteries significantly enlarged (Figure 2A, 2B). CT brain perfusion showed marked cerebral hypoperfusion on the left side mainly ACA and MCA (Figure 3). Considering her symptoms and imaging, she was thrombolysed with alteplase (0.9mg/ kg) and started on dual antiplatelets and statins (after 24 hours of thrombolysis). At the time of discharge, she could walk as her power regained to 4/5 on right limbs and was able to do her routine daily work. She could even have two-way conversations.

## DISCUSSION

Bilateral internal carotid artery occlusion is an extremely rare entity. It carries a grave prognosis.^5^ It accounts for 0.4% of strokes (transient ischemic attacks or patients with completed stroke).^6^ In another study, it was found that the annual stroke rates in patients with bilateral internal carotid artery occlusion ranged between 0% to 13%.^7^ Although it is rarely seen clinically, bilateral internal carotid artery occlusion patients can be expected to suffer serious consequences due to fatal ischemic stroke.^8^ It was reported that the overall mortality of 21 Bilateral internal carotid artery occlusion patients followed up for 1-11 years (average 6 years) was 52%.^1^

In bilateral internal carotid artery occlusion the collateral circulation comes from the vertebrobasilar system with cross-filling of the middle cerebral artery via the circle of Willis, an external carotid/ ophthalmic anastomosis, or a combination of the two.^9^ Atherosclerosis is considered the most significant mechanism of arterial occlusion/stenosis.^10,11^ Similarly hypoperfusion and embolism are the mechanisms considered for ischemic stroke in bilateral internal carotid artery occlusion where decreased cerebral perfusion and insufficient collateral circulation may increase the impact of embolism by impairing washout in hypoperfused areas resulting in symptomatic brain ischemia.^11-13^ Hypertension, hyperlipidemia, diabetes mellitus and smoking are the main risk factors contributing to atherogenesis and increasing the risk of artery stenosis/occlusion.^14,15^

The proper treatment of bilateral internal carotid artery occlusion remains controversial, a metaanalysis conducted by Mylonas SN, et al. revealed no significant difference in therapeutic effect between medical therapy and revascularization.^16^ So far, the clinical data of medical or surgical bypass management in bilateral internal carotid artery occlusion is also limited. Persoon S, et al. observed that 57 patients with Bilateral internal carotid artery occlusion treated by medical therapy achieved a better prognosis than did the surgical group.^7^ Patients with conservative medical management have survived with risk of recurrence of stroke.^7,9,20^ However, there is no case report suggesting a better outcome after IV thrombolysis in bilateral internal carotid artery occlusion.^17-19^ In our case the patient presented to our centre within three hours of the onset of right-sided weakness. We thrombolysed the patient with intravenous alteplase. Weakness improved significantly and the patient was discharged after a week of hospital stay.

Bilateral internal carotid artery occlusion is a rare and serious vascular disease. It often results in a fatal ischemic event. Here, we report a bilateral internal carotid artery occlusion patient who presented with symptoms of ischemia. The collateral compensation came from the vertebrobasilar system by cross-filling the circle of Willis. The patient presented in our centre within three hours of the start of the symptoms and we thrombolysed the patient with alteplase in the conventional dose of 0.9mg/kg. The patient improved significantly and was able to walk home. Due to the limited cases so far, the proper treatment of bilateral internal carotid artery occlusion remains controversial. Furthermore, long-term observation is necessary to obtain a better understanding of its therapeutic effect once surgical or conservative treatment is selected.
